# Eye Movements and Fixation-Related Potentials in Reading: A Review

**DOI:** 10.3390/vision4010011

**Published:** 2020-02-03

**Authors:** Federica Degno, Simon P. Liversedge

**Affiliations:** School of Psychology, University of Central Lancashire, Marsh Ln, Preston PR1 2HE, UK; SPLiversedge@uclan.ac.uk

**Keywords:** reading, eye movements, event-related potentials, fixation-related potentials

## Abstract

The present review is addressed to researchers in the field of reading and psycholinguistics who are both familiar with and new to co-registration research of eye movements (EMs) and fixation related-potentials (FRPs) in reading. At the outset, we consider a conundrum relating to timing discrepancies between EM and event related potential (ERP) effects. We then consider the extent to which the co-registration approach might allow us to overcome this and thereby discriminate between formal theoretical and computational accounts of reading. We then describe three phases of co-registration research before evaluating the existing body of such research in reading. The current, ongoing phase of co-registration research is presented in comprehensive tables which provide a detailed summary of the existing findings. The thorough appraisal of the published studies allows us to engage with issues such as the reliability of FRP components as correlates of cognitive processing in reading and the advantages of analysing both data streams (i.e., EMs and FRPs) simultaneously relative to each alone, as well as the current, and limited, understanding of the relationship between EM and FRP measures. Finally, we consider future directions and in particular the potential of analytical methods involving deconvolution and the potential of measurement of brain oscillatory activity.

## 1. The Timing Conundrum of Eye Movements and Event-Related Potentials

Decades of research recording eye movements (EMs) have revealed much about the processes that underlie written language comprehension and their temporal course [1,2]. One of the most important findings in the EM literature is that reading processing is fast and highly incremental [3]. Readers construct an incremental interpretation of the sentence, roughly on a word-by-word basis, as successive fixations are made along a sentence (e.g., [4,5]). Fixations are short periods of time, which on average last approximately 250 ms, during which information associated with the currently fixated word in the fovea, and to some extent with the upcoming word in the parafovea, is extracted and processed [1,2,6]. It is widely accepted that fixations reflect online cognitive processing [7,8], as the duration, and to a certain degree the location, of each fixation is determined by a number of cognitive factors (e.g., word frequency [9,10]; word predictability [11,12]). By implication, a sequence of processes occurs during each eye fixation. The sequence must include, as a minimum, transmission of the signal associated with the written word from the retina to the visual cortex, visual encoding, initiation of word identification, and programming of the next eye movement [13].

The large amount of robust evidence reflecting the rapid time course of reading from the EM literature however is in contrast with the likewise robust and compelling evidence of effects with a later time course reported in the ERP literature [3]. ERPs are EEG signals recorded at the scalp (with no measurable conduction delay between scalp potential and underlying source activity [14]) and time-locked to specific events [15]. They reflect postsynaptic potentials generated by populations of neurons active in synchrony, spatially aligned, and with the same direction of current flow [16,17]. A great number of studies recording ERPs during written language comprehension have consistently shown electrical signatures of linguistic processing in late time windows, associated with the N400 (between 300–500 ms after stimulus onset [18,19], although onsets are observed also from 200–250 ms after stimulus onset, e.g., [20]) and P600 (between 500–800 ms [21]) components.

ERP measures have been traditionally recorded using the rapid serial visual presentation paradigm (RSVP; e.g., [22]) in experiments focusing on the identification of individual words within or without a sentence context. In these traditional ERP experiments, one word at a time is displayed in the center of the screen, and blank screens are presented in between words. Each word is typically displayed for between 400–1000 ms, that is, for a period much longer than the average fixation duration in natural reading. Under these circumstances, ERP waveforms can be considered as a single stream of data corresponding to the cognitive processing associated with a single word during the entirety of the exposure period. Thus, finding that linguistic manipulations modulate late ERP components, and therefore, that observable effects associated with higher levels of cognitive processing occur at time points beyond the duration of an eye fixation, might seem unremarkable. However, we know from the EM literature that during natural reading, late time windows associated with these components are periods of time when the eyes have already moved to the next word, and identification of that word may remain underway, or indeed, may have been completed. Processing of a printed word in context, as reflected in EM measures, is determined by processing of both the individual word and integration of that word with the syntactic structure and the semantic representation of the sentence context constructed up to that point. Thus, observing modulation of ERP components that occur in relatively late time windows might reflect processing associated with fixations on words downstream in the sentence from the word in relation to which it was initiated. The important point to note here is that making a single long fixation on a word, or multiple refixations on a word, or even making multiple fixations after having left that word, might reasonably reflect qualitatively different aspects of cognitive processing [23].

Combining the on-line recording of EMs under natural reading conditions, and the real-time ms-by-ms recording of ERPs has great potential to unravel the nature and time course of the processes underlying reading [24,25]. Investigating neural correlates of foveal processing when both foveal and parafoveal information are available to the reader could lead to at least three potential scenarios. If effects associated with a linguistic manipulation are observed in late time windows, this would support the more traditional results that exist in the ERP literature and provide some evidence that a certain amount of time needs to pass for a linguistic manipulation to show a measurable effect in electrical brain activity. That is, cortical processing associated with word processing might outlast the fixational pause and behavioural response [26,27]. In contrast, if effects are observed exclusively in early time windows, this would bring into question the validity of the traditional ERP effects and raise the possibility that the nature of the paradigm used might affect the nature of the differences. For example, it might be possible that the foveal effects observed in previous traditional ERP studies are the result of a delay in processing due to the unavailability of parafoveal information, or due to differences in the deployment of attention under these experimental conditions relative to natural reading. Finally, if effects are observed in both early and late time windows, this would provide evidence that modulation of early ERP components might reflect cognitive processing associated with the identification of a fixated word, while later ERP components might also reflect similar processes as well as cognitive processing related to the integration of that word with its sentence context (e.g., semantic and syntactic processing).

## 2. A Tool to Discriminate between Theoretical Accounts

A large body of evidence from EM studies has demonstrated that readers not only process the word they are fixating, but also the upcoming word in the parafovea, that is to the right of fixation in alphabetic languages such as English (see [1,2] for reviews). Pre-processing of parafoveal information facilitates subsequent foveal processing of that word, and this contributes significantly to the rapid rate at which we read [6]. While in the EM literature parafoveal processing has been investigated for over 40 years (e.g., using gaze contingent paradigms [28,29]), parafoveal processing could not be investigated with ERPs until very recently, due to the nature of the paradigms being used. To reduce contamination of the EEG signal by EM artefacts and component overlap, words were presented one-by-one in the middle of the screen (e.g., [30,31,32]), or in the periphery away from central vision (e.g., [33,34,35]), such that normal parafoveal processing in reading could not occur. Recently, new paradigms such as the RSVP-with-flanker-word presentation method have been developed to address this issue (e.g., [36,37,38,39]). According to this paradigm, sentences or lists of words are presented word-by-word in the centre of the screen, with the preceding and following word(s) of the sentence, or of the word list, displayed laterally. The lateral presentation of the preceding and following words allows for parafoveal processing of the upcoming word, as in natural reading. However, in this situation, participants are required to keep their gaze on the centrally presented word, and not to make any eye movements. It is well documented that the allocation of attention and saccadic eye movements are most often tightly yoked (e.g., [7]), and for this reason, when participants are not required to plan and execute a saccade to the right, there are strong a priori grounds to anticipate that attentional allocation will not proceed in the same way as during natural reading. Thus, although ERPs have the potential to offer insights into the fine-grained timeline of parafoveal processing, and of foveal processing when parafoveal information is available, the experimental paradigms being used might not permit ready investigation of these issues. In this regard, co-registration of eye movements and brain potentials offers a methodological advance, and the possibility of investigating important theoretical questions that could not be addressed through the use of one of the two techniques alone.

Parafoveal processing is at the heart of the historical debate between serial versus parallel models of saccadic control in reading. The extent to which lexical processing of a parafoveal word is carried out during processing of the currently fixated word, and the temporal course of the lexical processing of the parafoveal word, are important issues in the reading literature. According to the serial accounts (e.g., E-Z Reader model [40,41,42]), words are fully lexically identified one word at a time. Extraction of information from the word in the parafovea initially occurs when attention is shifted to the parafoveal word but whilst the eyes remain fixating the word in the fovea. From a serial perspective, parafoveal pre-processing is largely limited to visual, orthographic and phonological properties of the word in the parafovea. Lexical processing of the upcoming word is initiated whilst it still lies in the parafovea. The initiation of parafoveal lexical processing occurs only after the reader is assured of the familiarity of the currently fixated word [43,44]. In contrast, advocates of parallel models of reading (e.g., SWIFT model [45,46]; see also OB1-reader, [47]) argue that more than one word is lexically processed at a time, with the degree to which parafoveal words are processed being determined by a number of factors including the word’s frequency and where it lies within the graded attentional window. According to parallel models, extensive (lexical) pre-processing of the word in the parafovea is expected during processing of the currently fixated word (e.g., [45,46]).

Co-registration of eye movements and brain potentials might allow for discrimination between theoretical accounts, as manipulation of characteristics of the word in the parafovea should have a different temporal influence according to the different models. Indeed, by time-locking the ERPs to the fixation onset of the word in foveal vision, it is possible to examine whether and which characteristics of the parafoveal word might be extracted and processed during processing of the foveal word. If processing of the parafoveal word is initiated only after the foveal word is identified, then we should observe an effect in time windows that follow latencies associated with foveal lexical processing. Alternatively, if visuospatial attention is distributed across multiple words but lexical access proceeds serially, manipulation of visual, orthographic and phonological properties of the word in the parafovea might elicit an effect in the time windows associated with foveal lexical processing, while manipulation of higher level linguistic properties of the parafoveal word might produce an effect in later time windows. Both scenarios would provide evidence in support of serial models of saccadic control in reading. In contrast, if lexical processing proceeds in parallel across multiple words, manipulation of the higher-level linguistic characteristics of the parafoveal word might show an effect in the same time windows associated with lexical processing of the currently fixated (foveal) word. Furthermore, whether word position coding is flexible and expectation driven (as in the OB1-reader model; Snell et al., 2018 [47]) might also be tested with co-registration.

It is possible that serial and parallel models are two extreme accounts, and new theoretical frameworks might be able to better explain the existing empirical data (e.g., see the Multi-Constituent Unit account advocated by Zang [48] in the current Special Issue). Despite this, however, given the more fine-grained nature of the FRPs, as recorded with experimental paradigms that allow participants to freely read and make saccadic eye movements, and time-locked to specific oculomotor events, co-registration can be adopted to potentially shed light on this debate.

## 3. Phases of Co-Registration Research

### 3.1. Pioneering Co-Registration Studies

The idea of using a single technique to record eye movements and brain potentials has been developed over years of pioneering research. As early as in the 1950s, researchers investigated the existence of brain responses associated with EMs (see [49]). These studies revealed the existence of a sequence of components associated with saccadic EMs [50,51]. First, a presaccadic slow negative waveform has been reported, starting up to 1s before onset of a voluntary saccade over posterior frontal areas, and then extending over parietal areas, being maximal over the vertex [52,53,54,55], which some hypothesised to be similar to the ‘readiness potential’ [55]. Following this, a presaccadic slow positivity, also known as the antecedent potential, was observed between approximately 30–300 ms prior to saccade onset. This effect occurred primarily over occipito-parietal areas, but also over frontal areas of the scalp (e.g., [53,54,56,57]). This slow positive wave was found to be associated with processes that precede saccade execution, such as saccade planning and shifting of attention towards the next saccade target (e.g., [54,56,58,59]). Next, a biphasic wave shape (first negative and then positive), also called the presaccadic spike potential, was observed at saccade onset (with a sharp positive potential approximately 10–40 ms prior to saccade onset [54,55]) caused by the contraction of extra-ocular muscles associated with saccade execution. This potential, positive over centro-parietal areas of the scalp contralateral to the direction of the next saccade, and negative ipsilateral to the direction of the next saccadic EM, was present regardless of light or dark visual conditions [60,61], and modulated by saccade size and direction [62,63,64]. In addition, a positive response was also observed, originating in the visual cortex of awake individuals about 80–100 ms after fixation onset [51,65,66] in response to changes in the retinal image that accompanied the saccadic EMs [52,67]. This visually evoked response, labelled ‘lambda wave’, was observed when saccadic EMs were required [49,65], appeared to be modulated by physical properties of the stimulus (e.g., luminance and spatial frequency [68,69,70]), was not detected in darkness [49], and was considered to be associated with uptake of visual information [61].

However, it was in the 1980s that the first attempts to concurrently record EMs and ERPs were made in order to understand the cognitive processes that occur during reading. Marton and colleagues [33,34,35,71] conducted a series of ground-breaking experiments time-locking ERPs to fixation onsets (labelled ‘saccade-related potentials’, SRPs, due to the focus being on saccade offsets). In their experiments, participants were asked to move their eyes to a word presented in the periphery of the visual field, which was located about 20 degrees to the left or to the right of either the midline point of the screen [33], or the margin of a new row of text [34,35]. This approach was adopted to study reading under conditions that approximated natural reading, while keeping saccade amplitude constant and while controlling for the direction of the saccades, with the assumption being that ocular artifacts associated with left and right saccades cancel out EOG contamination of the waveform during averaging [54,72]. Marton and Szirtes found that execution of EMs produced an advantage both in the peak latencies of the SRPs (compared to the visual evoked potentials, VEPs) and in the mean reaction times [33], suggesting that differences can be observed depending on the naturalness of the task. Furthermore, for the first time they presented entire sentences on the screen (over multiple rows of text) and manipulated the final word of the sentence such that it could be congruous or incongruous with the previous context [34,35]. In addition to the presence of a more pronounced negative deflection for incongruous final words relative to congruous words (the established N400 component [30,31,73]), their findings also revealed significant differences between the two conditions at approximately 110–160 ms from fixation onsets, with SRPs being more negative for incongruous compared to congruous final words over frontal and central regions of the scalp [35]. These differences were observed simultaneously with the appearance of the lambda wave (peaking at approximately 130 ms after saccade offset over the occipital region of the scalp). Because the lambda wave was thought to reflect analysis of the physical features of the word, and the N400 component was thought to be associated with lexical access, the authors concluded that their findings represented evidence for an effect of sentence context before lexical access.

The theoretical questions investigated and the paradigm used (i.e., free reading of natural sentences) make these studies of relevance to the present discussion. However, a severe limitation was the very low spatial accuracy in determining eye fixations. EMs were measured via electro-oculogram (EOG) channels placed around the eyes, which is not an optimal method for measuring eye gaze. Thus, although subsequent efforts were made to improve this initial approach (e.g., [74]), research moved towards experimentation recording EMs with high-precision eye tracking devices which were being developed in the meantime (although see [75,76] for recent examples of reading studies using EOG channels).

### 3.2. Separate Recording of Eye Movements and Event-Related Potentials

In the 1990s, the scientific community became increasingly aware that comparing results from studies conducted with EMs and ERPs investigating the same theoretical question could provide a more complete understanding of the processes underlying reading [77]. However, the idea of concurrently recording EMs and ERPs was still considered to be out of reach. Two main issues were considered problematic: the disruption of the EEG signal caused by the ocular artifacts (saccades, blinks, etc.), and the component overlap across successive fixations [77,78]. Thus, a second phase of co-registration research was characterised by the comparison of EM and ERP data obtained from two separate experimental sessions [77,78,79,80]. Typically (except for [79]), in an EM experiment, sentences were presented normally while EMs of one group of participants were recorded. In a separate ERP experiment, testing different participants, target words, either within a contextual frame, or presented in isolation, were presented word-by-word according to the RSVP paradigm while the EEG signal was recorded. Although this approach was limited in a number of respects, the work raised a number of important theoretical questions.

Raney and Rayner [77] first compared converging results from two different existing studies ([81] for the EM data; [82] for the ERP data) that investigated the nature of changes in processing associated with rereading (i.e., when text was read for the second time). In these experiments, participants were required to read and remember as much text as possible for later recall. The EM data showed that multiple aspects of reading behaviour were facilitated during rereading (e.g., duration and number of forward fixations was reduced, amplitude of forward saccades increased, number of regressive fixations fell), and that the facilitation associated with the second reading affected high and low frequency target words similarly. The ERP data showed that changes in rereading behaviour were likely due, on one side, to decreased perceptual and comprehension demands (denoted by increased N1-P2 amplitudes), and on the other side, to increased memory demands (as suggested by decreased P300 amplitudes). Thus, converging evidence from EM and ERP data supported the theoretical view of the existence of different stages of processing during reading. However, Raney and Rayner did also report instances of diverging EM and ERP results. Different findings were observed in two experiments investigating processing of a target word that was related or unrelated to the preceding sentence context ([83] for the EM data; [84] for the ERP data). The EM data showed that facilitation occurred only when both the subject noun and verb were related to the target word. In contrast, ERP data showed facilitation when both or only one of either the subject noun or the verb were related to the target word. Although Raney and Rayner did not discuss in detail the diverging results, the paper raised an important issue concerning the relationship between EM and ERP measures. This relationship was later investigated by Dambacher and Kliegl [78]. These authors compared EM and ERP results from two separate experiments in which two different groups of participants were required to read the same sentences for comprehension ([85] for the EM data; [86] for the ERP data). The aim of the study was to investigate whether both EM and ERP measures (i.e., fixation durations and N400 amplitudes respectively) were modulated by the same word properties, and by implication, by common mechanisms associated with word recognition. They found that longer single fixation durations were correlated with more negative N400 amplitudes on the corresponding word, a relationship accounted for by both word frequency and word predictability, as well as by the predictability of the upcoming word. In addition, more negative N400 amplitudes were associated with longer single fixation durations on the following word, and this relationship was accounted for by word frequency. Thus, their findings confirmed that both EM and ERP measures were similarly modulated by word frequency (considered a bottom-up variable) and word predictability (considered a top-down variable), and, as a consequence, by at least one common stage of processing.

Taking advantage of these complementary methods, Ashby and Martin [79] also compared EM and ERP results. Aiming to investigate the nature of prelexical phonological processing, they used a boundary-change lexical decision task for the EM experiment, and a masked priming semantic judgment task for the ERP study. In both experiments, isolated target words were presented, preceded by a partial word parafoveal preview (in the EM experiment) or prime (in the ERP experiment). Both were comprised of a syllable congruent or incongruent with the initial syllable of the target word. Shorter fixation times and more positive ERP amplitudes between 250–350 ms were observed for the syllable congruent condition compared to the syllable incongruent condition. These converging results provided support for the view that activation of prelexical phonological representations includes activation of prosodic (i.e., suprasegmental) information, such as syllable information, that is not encoded in the writing system. In addition, they suggested a role for memory in the activation of suprasegmental information, since preserving congruent syllable information in memory during a saccade, or during a backward mask, facilitated word recognition.

Although the nature of the relationship between EM and ERP measures remains unclear and it has not yet been determined whether different results might be explained by differences in the specific paradigm used, the three studies [77,78,79] showed that considering converging, as well as diverging, EM and ERP results offers potential benefit for our understanding of reading, and of human cognitive processing more generally.

In this second phase of co-registration research, another important issue was investigated, namely, sensitivity of early ERP components to lexical manipulations. Sereno, Rayner, and Posner [80] investigated timing discrepancies for effects thought to index lexical access (e.g., word frequency [87]) between methodologies, that is, as reported in independent EM and ERP studies. They argued that it was not self-evident why experimenters should investigate relatively late ERP components, such as the N400, by default when investigating aspects of lexical processing. An alternative, arguably more plausible, approach might be to attempt to account for lexical effects within a more typical EM time-line (i.e., considering lexical processing effects within 250 ms from fixation onset). By recording EM and ERP measures in response to the same target words (within a sentence in the EM experiment and in isolation in the ERP experiment) their study showed effects of lexicality as early as 100 ms from stimulus onset (on the P1 component), effects of word frequency starting at 132 ms from stimulus onset (on the N1 component), and effects of word regularity (in terms of spelling sound correspondence) as early as 164 ms from stimulus onset (on the P2 component). Thus, as they argued, their study did show lexical effects on early ERP components, although the two groups of participants were engaged in quite different tasks in the two experimental sessions: silent reading for comprehension in the EM experiment, and lexical decision task in the ERP experiment. This study emphasised the need for more research on the early ERP components when investigating the cognitive processes underlying reading, which had been largely ignored up to that point.

It was during this period that the idea of a single technique that would simultaneously combine EMs and ERPs was formalized [24,25], thereby initiating the modern conception of co-registration research. It was evident that the existence of electrophysiological signatures associated with specific cognitive processes within an eye fixation had the potential to reveal a more precise timing of the sequence of processing and to offer insight into the nature of pre-lexical, lexical, or post-lexical processes underlying reading.

### 3.3. Simultaneous Recording of Eye Movements and Fixation-Related Potentials

Despite the existing challenges associated with simultaneously recording EMs and ERPs (see [26,88,89,90,91] for a discussion), joint efforts from the international research community, alongside technological advances (see [92,93,94,95] for a discussion) and advances in computational algorithms used for the correction of ocular artifacts [26,96,97,98,99], have allowed for a third, ongoing, phase of co-registration research to become possible. These studies have involved simultaneously recording EM and ERP data from the same group of participants, reading the same stimuli, and performing the same task. This has ensured an exact correspondence between EM and ERP data under identical experimental testing conditions in the same individual. In this approach ERPs are referred to as fixation-related potentials (FRPs; or EFRPs, e.g., [100]), as the time-locked events of interest are fixation onsets on particular words in a trial. Note, though, that ERPs can also be time-locked to saccade onsets, in which case we speak about saccade-related potentials (SRPs; see for example [91] for a discussion). A variety of different paradigms have been used under such testing circumstances, for example, free reading of pairs of words, lists of words, sentences or even paragraphs, during which participants make saccadic EMs. Via these paradigms, it is possible to shed light on the neural correlates of not only foveal processing, but also parafoveal processing, an important aspect of reading that was not possible to investigate in experiments conducted in the first two phases of co-registration research.

A complete list of studies involving simultaneous recording of EMs and FRPs is presented in Table 1. Note that we include in Table 1 only co-registration experiments that have investigated reading through the analysis of EMs and FRP components, in which participants were allowed to make at least forward eye movements in each trial, and in which eye movements were recorded with an eye tracking system. Studies using co-registration but analysing oscillatory brain activity time-locked to fixation onsets on particular target words [101,102,103,104,105] will be discussed only in relation to future directions.

**Figure 1:**
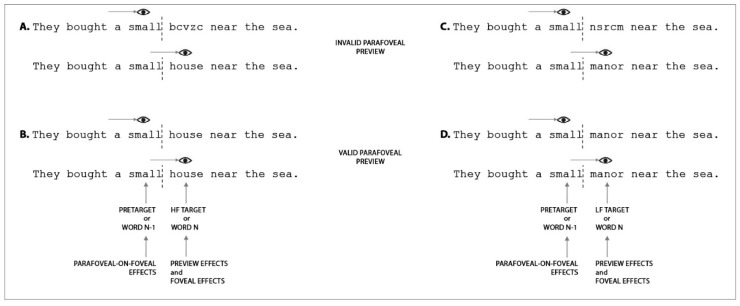
Illustration of example stimuli and investigated effects. The image shows example sentences presented according to the boundary paradigm [28]. An invisible boundary is placed at the end of the pretarget word (i.e., ‘small’). When the eyes fixate the pretarget word, a preview is displayed in the parafovea (i.e., ‘bcvzc’, ‘house’, ‘nsrcm’, ‘manor’). When the eyes cross the invisible boundary, the target word is displayed (i.e., ‘house’, ‘house’, ‘manor’, ‘manor’). Panels A and B differ from panels C and B in the frequency with which the target words ‘house’ and ‘manor’ occur in the English language, such that ‘house’ is a high frequency (HF) word, ‘manor’ is a low frequency (LF) word. In addition, panels A and C differ from panels B and D as the preview stimulus is an invalid preview in the first two panels, but a valid preview for the other two panels. That is, in panels A and C, a string of random letters is presented in the parafovea, and this string does not share many features with the target word (i.e., bcvzc’–‘house’, ‘nsrcm’–‘manor’). Instead, in panels B and D, preview and target words are identical (i.e., ‘house’–‘house’, ‘manor’–‘manor’). Parafoveal-on-Foveal (PoF) effects are examined by time-locking EM and FRP data to the onset of the first fixation on the pretarget word. Thus, researchers examining PoF effects, compare the effect that the different parafoveal previews (i.e., ‘bcvzc’, ‘house’, ‘nsrcm’, ‘manor’) have on the processing of the pretarget word (i.e., ‘small’) that is currently being fixated. Preview effects are studied by time-locking EM and FRP data to the onset of the first fixation on the target word. Here, researchers compare the effect that the different parafoveal previews (i.e., ‘bcvzc’, ‘house’, ‘nsrcm’, ‘manor’) have on the processing of the target word (i.e., ‘house’ or ‘manor’) when it is subsequently fixated. Foveal effects are investigated by time-locking EM and FRP data to the onset of the first fixation on the target word and comparing how the characteristics of the stimulus in fovea (i.e., a HF word ‘house’ vs. a LF word ‘manor’) affect processing of that word.

Below we present three tables (Table 2, Table 3 and Table 4) in which we summarize every experiment that has been conducted to investigate parafoveal and foveal processing, presenting, and in some cases manipulating, information in the parafovea under co-registration conditions (see Figure 1 for a visualization of the investigated effects).

Table 2 provides a summary of the results associated with effects derived from parafoveal manipulations measured during fixation of the pretarget word. In these studies, the parafoveal word was manipulated, and the EM and FRP data were time-locked to the fixation onset of the pretarget word, thus allowing for investigation of parafoveal-on-foveal (PoF) effects. As discussed in Section 2 of this review, the time course of PoF effects is crucial in the context of the debate between serial and parallel models. Whether lexical characteristics of the parafoveal word are extracted after or during lexical processing of the foveal word pertains directly to serial or parallel accounts, and whether visuospatial attention and processing operates in a focused or a distributed manner. Note, though, that whilst early effects might be clearly associated with processing of the parafoveal word during fixation on the pretarget word, FRPs associated with later time windows might be contaminated by activation associated with foveal information processed during subsequent fixations on the following (target) word, or refixations on the same (pretarget) word, implying that effects in these later time windows require very careful analysis and consideration.

Table 3 provides a summary of experiments demonstrating effects at the target word when it was manipulated in some way whilst presented in the parafovea. The effects associated with this manipulation were measured when both EMs and FRPs were time-locked to the initial fixation onset on the target word. These studies aimed to examine the influence that the pre-processing of an upcoming word in the parafovea exerts on the processing of that word when currently fixated. In the EM and reading literature this effect is generally called the preview benefit effect and it is usually investigated using the boundary paradigm [28]. In the boundary paradigm, the target word is replaced by a preview string and an invisible boundary is placed immediately prior to the target word. In this way, when the eyes are fixating the pretarget word, parafoveal processing of the preview occurs. However, when the eyes cross the boundary, the preview is replaced by the target. If efficient parafoveal processing of the preview occurs, and the preview is related in some way to the target, then if the characteristic that is shared between the target and the preview is processed parafoveally, there will be a processing benefit at the target (though see [124,125,126] for discussion of parafoveal preview cost relative to parafoveal preview benefit). Thus, by systematically manipulating the characteristics of the preview in relation to the target word, it is possible to make inferences regarding which properties of the preview were parafoveally pre-processed prior to direct fixation. For example, if a parafoveal preview and target word are semantically associated, then if the preview is lexically processed, semantic facilitation of the target word should be observed upon fixation. Alternatively, if parafoveal pre-processing is limited to visual and orthographic (but not lexical) properties of a preview, then no facilitation at the target should occur. Again, investigations of this kind seek to discriminate between serial or parallel models of reading.

Table 4 provides a summary of the existing findings observed for foveally triggered effects as measured during fixation of the target word. That is, in these studies variables that affect processing of a word from (at least) its initial fixation onward and their time course are examined. Thus, the effects observed in these co-registration experiments should closely match with the results observed in traditional ERP studies. However, knowing that the time course of word processing is affected by the availability of parafoveal information, this research might investigate fairly directly the timing conundrum that exists in relation to EM and ERP effects (see Section 1).

The information that is provided in the tables is comprehensive. Space limitations preclude an extended discussion of all the aspects of the studies that are reviewed in these tables, however, evaluating all the information together leads us to form two conclusions.

First, studies have consistently observed two neural correlates of identity preview benefit. The first neural correlate is such that between 200–280 [110,114], between 140–200 and 200–300 [117], and between 120–300 [120,121], N1 amplitudes are more positive for identical previews compared to invalid previews, largely over occipito-parietal and temporal areas of the scalp. The only study which failed to find such an effect [115] involved a methodological difference such that linked mastoids were used as an offline reference (see [39] for discussion). The second neural correlate of identity preview benefit is observed between 360–400 ms, when valid previews elicit more positive amplitudes than invalid previews over central sites of the scalp [102,110,115], and between 300–500 ms, when valid previews elicit more negative amplitudes than invalid previews over occipital areas of the scalp [121]. This late preview effect on the N400 component was not observed in Degno et al. [120], indicating that this might be related to naturalness of the reading task or to baseline choices. The eye movement results also mapped onto the FRP data. Overall, and to date, this identity preview effect represents the most robust and well documented effect in the co-registration literature, having been investigated and demonstrated in word list reading experiments, prime-target pair experiments, as well as in natural reading experiments. Current understanding of the neural correlates of the identity preview benefit suggests that the latency range associated with the N1 component might reflect the period of time when efficient orthographic and/or phonological processing occurs, because orthographic and/or phonological characteristics of a word are correctly activated based on parafoveal information (see [110,121]). The effect observed on the N400 component might likely be a consequence of the effect shown on the N1 component, suggesting that when orthographic and/or phonological processing is disrupted, later cognitive processing (likely lexical or semantic processing) is also slowed down and disrupted.

The second conclusion is that the literature is limited, the findings are mixed and sometimes inconsistent. This leads us to the view that much more experimental work using these techniques is necessary to allow us to identify those effects that occur with reliability and that are robust across studies. Fairly consistent results have been observed between EM and FRP measures for PoF effects of parafoveal preview [106,109,120], preview effects of predictability [26,108,113], frequency [113,117,120], and type of preview [110,114,117,120], foveal effects of text type [111], inter-letter spacing [118], repetition [107,110,112], word predictability [26,104,108,113], syntactic and semantic violations [116,119] and foveal load [114]. However, inconsistencies in EM and FRP results have been observed for PoF effects of semantic relatedness [106,108,109,110,115], preview effects of semantic relatedness [110,115], foveal effects of semantic relatedness [108,110,115] and word frequency [113,120]. It remains the case, though, that co-registration investigations of aspects of reading are in their formative stages (with some effects being investigated in single studies only) and a greater body of experimental data is a necessity before firm conclusions may be formed as to the kinds of experimental manipulations that regularly and consistently produce FRP effects of specific kinds. Given the current limitations with respect to the restricted empirical investigations employing the co-registration method alongside the relatively small number of data sets, to us, it feels appropriate to place emphasis on only those effects that appear strongest across studies at this point.

## 4. Are FRP Components Reliable Correlates of Cognitive Processes in Reading?

**Figure 2:**
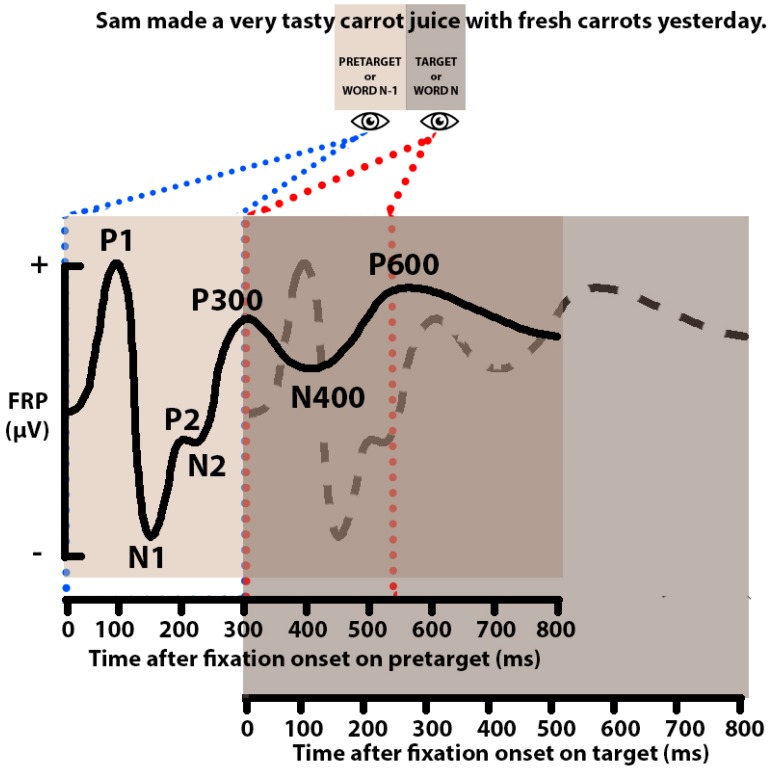
Here we offer a stylized characterisation of deconcolved waveforms that might (ideally) be revealed if deconvolution processes were applied successfully to an average FRP data stream recorded across two successive fixations made on the word “carrot” and then on the word “juice” as the sentence “John made a very tasty carrot juice with fresh carrots yesterday” was read. The solid black line represents the waveform and components (i.e., P1, N1, P2, N2, P300, N400, P600) that result from processes associated with the fixation on the pretarget word, and these have been separated from the waveform and components (dashed line) associated with the fixation on the target word. Note that the actual waveform that would be recorded during the experimental trial would be a convolved signal comprised of multiple waveforms deriving from fixations on the word(s) prior to the pretarget word, the target word (and potentially) the posttarget word (‘with’ in the current example). For simplicity, we have not included the convolved waveform in this figure, but the overlapping portion of the two panels (time-locked to fixation onset on pretarget and target words) shows where that convolved signal would occur (for only pretarget and target words) if it had been illustrated. See Ehinger and Dimigen (2019) [130]) for a discussion of deconvolution and its mathematical properties.

As discussed in the previous section, existing co-registration studies demonstrate a series of prominent FRP components (e.g., P1, N1, N400, etc.) that are elicited during reading tasks (see Figure 2). Understanding of what these FRP components might represent in relation to cognitive processing, at this stage, is currently developing. In this section, we briefly describe each component and consider the aspects of processing associated with reading with which each may be associated.

The earliest effect that we observe is a positive visually evoked response that originates from the occipital regions of the scalp with a peak at approximately 100 ms after fixation onset on a written word. This visually evoked response, once labelled ‘lambda wave’, is now considered the equivalent of the P1 component [26,110]. Indeed, both responses appear to be associated with the uptake of visual information [61], and to have a common neural generator in the visual cortex [127]. The P1 component seems to be modulated by the nature of parafoveal preview [120,121], as well as the visual form of the text [111].

The P1 component is followed by the N1 component, a negative potential which is largest over left occipital, parietal and temporal areas of the scalp, with a peak approximately 200 ms after fixation onset. Existing co-registration studies support the view that the N1 component is a time-window during which orthographic and/or phonological representations of a written word become activated (see effects of parafoveal preview on the N1 component [110,114,117,120,121]), and that the negativity in this latency range is increased when less effective orthographic and/or phonological pre-processing of parafoveal information occurs [114,120,121]. In addition, there is some evidence that effects of word frequency [117] and foveal load [114] also modulate the N1 latency. At a first glance, these timing effects might fit well with previous ERP studies that have shown word frequency effects on early components (e.g., [80,128,129]), and with the time-line constraints derived from EM effects [25]. However, currently, given the sparse evidence for such effects, and the failure to find these effects in sentence reading experiments [113,120], further investigation is needed.

Following the N1 component, a P2 component has also been observed in several co-registration studies [106,109,113]. This positive potential was observed first over anterior and central areas of the scalp between approximately 140–280 ms after fixation onset, being modulated by parafoveal semantic relatedness [106] and word predictability [113], and then later between approximately 200–280 ms over occipital scalp sites being modulated by parafoveal word form [109]. However, again, given the small number of studies to have found modulation of this component, the functional description of the P2 component currently remains unclear.

Another prominent FRP component is the well-established N400, a negativity observed over centro-parietal areas of the scalp. This FRP component appears to be modulated by both parafoveal and foveal manipulations. The parafoveal N400 component time-locked to the fixation onset on pretarget words is modulated by parafoveal preview type [120] and parafoveal semantic relatedness [108,115]. The N400 component time-locked to fixation onset on the target word seems to be modulated by parafoveal preview type [110,114,115,121], semantic relatedness [110,115], word predictability [26,104,108,113], as well as syntactic and semantic violations [116,119]. Observing modulation of this component during natural reading is noteworthy. In one respect, it suggests that there is a stage of processing in this time latency that is affected by linguistic manipulations regardless of the paradigm used, and therefore that the component might reflect aspects of processing that continue beyond a single eye fixation [27]. In another respect, it raises the interesting possibility that the same cognitive mechanism might underlie effects observed in both early and late components (e.g., the parafoveal preview effect observed on both the N1 and N400 components).

The N400 component is followed by the P600 component, a positive response with a widespread distribution (largest over centro-parietal sites), with a peak at approximately 600 ms from fixation onset. The P600 component time-locked to the fixation onset on target words appears to be modulated by parafoveal preview type [115], semantic relatedness [108,115], word predictability [104], as well as syntactic and semantic violations [116,119].

The existing findings suggest that FRP components that are observed in existing co-registration studies are quite comparable to components observed in more standard ERP investigations, and these appear to be elicited by similar experimental manipulations. Given this, it seems appropriate to argue that FRP components are as reliable electrophysiological correlates of cognitive processing underlying reading as those observed in more traditional (non-co-registration) ERP data sets. In addition, the ecological validity advantage of the FRPs over the traditional ERP methods allows for a more comprehensive investigation of the neural correlates of reading.

## 5. Do Co-Registration Studies add Value to Our Understanding of Reading?

In answering this question, we consider that there are at least two relevant perspectives, in that co-registration adds value (and here we mean in the sense of providing increased scientific insight) both to studies in which EMs alone are recorded, as well as to studies in which ERPs are solely recorded.

Let us first consider whether co-registration studies, potentially at least, offer greater insight than studies in which only EM data are recorded. In relation to this issue, note that EM studies investigating reading very often produce patterns of effects that are statistically robust with clear and predicted numerical effects. Furthermore, as our discussion of the existing co-registration literature above should elucidate, such clear EM effects are in stark contrast to patterns of effects in FRP studies that can frequently be mixed and much more difficult to interpret. Despite this, based on our assessment of this body of work, it is our view that co-registration studies do offer added value. The study by Degno et al. (2019) [120] might serve as an example to substantiate our claim. In their experiment, Degno et al. (2019) [120] obtained no significant difference in the EM data between X-string and letter string parafoveal preview conditions at the target word. The early first-pass reading EM results associated with both X-string and letter string parafoveal previews showed similar disruption to reading compared to the identity preview conditions (X-string vs. identity preview condition: difference of 41 ms in first fixation duration, 63 ms in single fixation duration; letter string vs. identity preview condition: difference of 41ms in first fixation duration, 57 ms in single fixation duration). Thus, on the basis of these data, there was no observable processing difference between the X-string and letter-string preview conditions. However, significant differences between these conditions were observed in the FRP data. Our interpretation of this result is that during the fixations (of numerically very similar duration), the nature of the processing that occurred in the brain produced an amount of disruption under each condition that was comparable, therefore increasing reading behaviour similarly. Yet, even though this was the case, it seems entirely reasonable to us to suggest that the disruption that did occur during these fixations may well have been qualitatively different in nature. If this suggestion is correct, then these results exemplify one way in which FRP data may add value by way of scientific insight, in that they offer the potential to discern between qualitatively different types of processing that each cause quantitatively comparable disruption to fixation durations.

Next, let us consider how co-registration as an empirical method may offer increased potential insight relative to studies recording EEG data alone. From an ERP perspective, it might initially be difficult to appreciate the potential of co-registration under free reading conditions, in that experiments of this kind add disruption to the EEG signal via ocular artifacts that arise due to saccadic EMs intrinsic to reading. However, let us briefly consider the studies that have directly compared results obtained under active (i.e., saccadic reading) and passive (i.e., RSVP or RSVP-with-flankers paradigm) reading conditions ([107,112,114,116,117]). These studies have shown several effects that have similar scalp distributions in both the passive ERP and active FRP settings (although see Niefind and Dimigen (2016) [117], where the parafoveal preview effect and preview frequency effect were observed only in the FRPs, and Metzner et al. (2016) [116], where sentence-final semantic violations elicited different voltages in the N400 latency range for the RSVP condition, and in both the N400 and P600 latency ranges in the FRP reading condition). However, and critically in relation to our argument here, in all these studies those effects were larger in size, were longer lasting and had an earlier onset in the FRP active reading conditions compared to the passive ERP reading conditions. For example, Kornrumpf and colleagues (2016)[114] observed a parafoveal preview effect between 230–250 ms for passive reading ERPs, but between 160–300 ms in FRPs under active reading conditions. We consider that although this difference in the onset of the effect is small, it is likely very important in that it might reflect a more advanced timeline associated with natural reading due to the rapid dynamics of attentional deployment in such circumstances. Clearly, it is the case that the time of processing has been an issue of central investigation in reading research, and given this, to us at least, any such differences are potentially informative regarding the precise nature of processing.

In addition, with co-registration it is possible to investigate neural correlates of specific and different aspects of oculomotor behaviour that might occur under different experimental conditions during reading ([116,122]). For example, Metzner et al. (2016) [116] separated trials in which participants did, or did not, make a regressive saccade in order to re-read previous parts of the sentence, and revealed different FRP effects associated with re-reading of syntactic and semantic violations. That is, whilst trials with regressions elicited effects on the N400 (for sentence-final syntactic and semantic violations) and P600 component (for sentence-medial and sentence-final syntactic and semantic violations), in trials without regressions effects associated with sentence-final syntactic and semantic violations elicited a sustained negativity only. In this way, again, FRP data offer potential for added value.

Finally, an area of reading research that has received little attention, but which may be fruitful for co-registration research, concerns the investigation of oculomotor planning per se. For example, we question whether there may be differential FRP signatures associated with alternative patterns of oculomotor activity. For instance, whether there is a different FRP ”signature” associated with a fixation prior to a progressive saccade relative to that for a fixation prior to a regressive saccade.

## 6. What is the Nature of the Relationship between Eye Movements and Fixation-Related Potentials?

Let us state at the outset that the currently limited number of studies mean that a completely clear perspective is not, at present, possible. Moreover, this is an extremely difficult question to tackle. Nonetheless, we do feel that it is at least necessary to consider aspects of those results that do currently exist that might inform an answer. For example, we consider it important, as we have already noted, that there are relationships and consistencies in EM and FRP data sets that are suggestive of common (potentially causative) links at the level of neural and cognitive processing. If the suggestion that such commonality across data streams is reflective of common aspects of psychological function, then such results are extremely encouraging in respect of the future value of the co-registration approach as a tool to further future understanding. At this stage, from our perspective, we see enough such effects in existing data sets to persuade us that this venture is worthwhile. All of this said, it remains the case that there are a number of situations when there are inconsistencies in effects across the two data streams. Furthermore, such inconsistencies may take the form of a particular effect in one data stream seemingly corresponding to multiple different effects in the other data stream. Alternatively, inconsistencies might even occur such that whilst an effect occurs in one data stream, there is no evidence of any corresponding effect in the other stream. If both EM and FRP data streams do reflect common causative neural and cognitive aspects of processing, then it is quite unclear why such inconsistency should arise. Again, at this stage, we feel it is prudent not to speculate to any great degree as to potential explanations for such data patterns. Instead, it seems likely that over time as results from co-registration studies proliferate, the patterns of consistency, as well as the patterns of inconsistency will develop and become substantiated, and at such a point downstream, we should then be better equipped to provide an answer to the question that we have posed.

Even though we are cautious in our interpretation of existing co-registration data set, based on our assessment of current data patterns, it appears that during natural reading, when a clear expectation is not met, as for example with predictability and semantic and syntactic violation effects, both behavioural and neural systems exhibit pronounced disruption. The disruption can be observed clearly and comparably in both the EM and FRP data streams, with counterpart “signatures” in both. In contrast, when subtler, less disruptive manipulations are adopted, such as word frequency and semantic relatedness manipulations, where processing may proceed relatively unhindered (i.e., without pronounced disruption), then it appears that less consistent counterpart effects appear in the two data streams. Thus, potentially, the degree of disruption (that is, both the magnitude of cognitive disruption, as well as the extent to which effects are statistically robust) that a manipulation causes to ongoing cognitive processing in reading may be the mediator of the degree to which comparable counterpart EM and FRP effects are observed. It is for future research to refine explanations of why EM and FRP recordings sometimes offer consistency in effects, and on other occasions inconsistency. The application of linked mixed models [131] to co-registration data might be a useful analytical tool to further our understanding of the relationship between EM and FRP measures.

A final point that we will consider concerns the contrasting manner in which measures of EM and FRP are used in the literature. Within the EM research community, different EM measures (e.g., first fixation duration, single fixation duration, gaze duration, etc.) are used to show the temporal course of an effect, and it is well documented that different EM measures can be influenced by the same experimental variable (e.g., [132]), and that the same EM measure can provide insights into different aspects of processing associated with reading [23,77]. Thus, the reason why different, and often numerous, EM measures are analysed and reported is because the entire time course of an effect is being considered such that claims can be made as to the point in time at which an experimental manipulation first exerted an observable influence on processing, as well as the duration (and arguably, nature) of its influence. There is a crucial and fundamental difference between the approach to measures in the EM and ERP literatures. Broadly speaking, EEG researchers favour an approach in which effects are directly investigated through the examination of specific components known to be modulated by a particular experimental variable (although see for example [128,133] for a ‘time course processing’ approach). Of course, current understanding has moved on from the idea that individual components within an EEG data set index particular cognitive functions, almost on a “one-to-one” basis (e.g., N400 as a language measure of semantic processing). Instead, it is now widely considered that the EEG data stream provides an electrophysiological index of activity in the cortical mechanisms underlying a mental operation (e.g., N400 as an index of processing in semantic memory of a range of meaningful stimuli across different modalities; [18]). However, despite this, it remains the case that many ERP studies focus on particular ERP components, and seek to demonstrate an influence of one or more experimental variables on that component. In contrast to the approach in EM studies where the continuity of processing over time is investigated, in ERP studies, often, “snapshots” in time are offered, and in this way, insight into the entire time course of the influence of an experimental variable may be missed. In our view, co-registration research offers an opportunity to bring the “time course of processing” approach to analysis that is often utilised in EM research to the analysis of EEG data sets. For example, existing co-registration studies have analysed a series of FRP components that are initiated and developed from fixation onset, and such studies do adopt more of a time course approach to their analyses. In addition, the adoption of raster plots that illustrate data acquired from all scalp sites within relatively extended time windows of analysis, also seems to offer insight into the nature of change in FRP data over time.

## 7. Future Directions

An important issue that faces co-registration researchers concerns how we might most effectively separate components that overlap temporally (i.e., from one fixation to the next) within the EEG data (e.g., [26,77,78,88,111]). Figure 2 presents a visual example of the issue. As can be seen, each fixation has associated with it a waveform containing a series of components that overlap with the waveform and components associated with the preceding and following fixations. In the figure, the composite waveform has been separated (on the assumption of the successful application of deconvolution) to illustrate how components associated with each successive fixation might be isolated and identified. It should be clear that without deconvolution, the composite waveform would have a form represented by a combination of the underlying waveforms (and this is not represented in Figure 2).

Progress has been made in developing approaches to deal with this issue (e.g., [130,134,135,136,137,138,139]), but the majority of the existing, published, co-registration experiments investigating reading do not report analyses that separate temporally overlapping components deriving from different fixations, and therefore, have not yet directly addressed this challenge (although see [119] for an assessment of spatial overlap of components and [122] for temporal overlap). Limiting the analysis to time windows in which the next fixation does not yet overlap (e.g., selecting only fixations with a minimum fixation duration and analysing the FRP components within those intervals; see Nikolaev et al., 2016 [91] for a discussion) does directly address this issue. However, this approach leads to a series of other important considerations (e.g., fixations that could reflect meaningful cognitive processes might likely be systematically excluded from the analyses), which make the deconvolution approach a much better solution to date.

New advances in techniques to deconvolve the EEG signal whilst at the same time controlling for covariation due to other effects have been developed (e.g., [130]). Loberg et al. [122] have taken the first step to try to adopt this approach to investigate school-aged children with slow or typical reading speed as they performed a natural reading task where a target word in each sentence was manipulated for length. Deconvolved FRPs were analysed, and saccade amplitudes were used as covariates. Loberg et al. did not provide comparative analyses of convolved vs. deconcolved data sets, however, Ehinger and Dimigen [130] did undertake such analyses (though their task involved face processing, not reading). These analyses indicated that whilst the main components and effects are observable in both types of analysis, the analyses of the deconvolved data appeared more tightly and definitively indicative of specific processes associated with experimental manipulations. We anticipate that researchers adopting co-registration to investigate reading will increasingly engage with these techniques into the future.

A second area that we see as offering future promise is the investigation of brain oscillatory activity. Increases and decreases in power in specific frequency bands are considered to reflect synchronization and desynchronization, respectively, of the underlying neural networks. To date, a very limited number of co-registration studies has investigated brain responses in the time-frequency domain during reading [102,103,104,105]. Based on published studies to date, two considerations emerge that may be of particular importance. First, Kornrumpf, Dimigen, and Sommer [102] have shown that analysis of these data might best suit investigation of more covert aspects of cognitive processing in reading, such as distribution/deployment of attention in relation to foveal and parafoveal processing. In this sense, analysis of brain oscillation might become a crucial tool to use in the context of distinguishing between different models of reading that specify how and when attention is allocated across words over fixations. At a more general level though, brain oscillations might also offer an opportunity to investigate induced (rather than evoked) meta-cognitive aspects of reading, such as task engagement, attentivity, dual-task costs, distraction effects, discourse coherence and text comprehension, as well as tasks demands (see e.g., [103]). Again, we must reiterate that the investigation of brain oscillatory activity is currently limited in silent reading research (see [140]), and to an even greater extent in co-registration research. It is for future research to demonstrate the true value of the approach.

## 8. Conclusions

The present review has provided an overview of the history and literature on co-registration of EMs and FRPs in reading, and discussed the potential of this methodology for providing novel scientific insight into the nature of processes underlying reading. The appraisal of the existing literature has allowed us to raise questions for consideration that we hope will challenge current understanding and stimulate debate concerning the neural correlates of cognitive processing in reading. We consider the theoretical assumptions that underlie the co-registration approach to be plausible, and that consideration of both data streams simultaneously provides additional, complementary value to the insights we can obtain by considering either data stream alone. However, current understanding of the relationship between oculomotor events and neural correlates of those events remains unclear. It appears crucial to conduct a significantly larger number of experiments with this methodology to develop our understanding of how neural and cognitive processes associated with oculomotor events relate to their FRP correlates in reading. The timing and nature of these correlates time-locked to specific words will be instrumental in the development of better specified and more comprehensive models of reading.

## Figures and Tables

**Table 1:** Co-Registration Studies Presenting Pairs of Words (+), Lists of Words (++), Sentences (*) or Paragraphs (**).

Study	Language	Participants	Paradigm	Task	Variables	Investigated Effects
BM2005 ^+^ [106]	French	Age range: 22–38 Average age: 27.0 Typical readers	Priming	Semantic association judgment	Parafoveal preview, Semantic relatedness	Parafoveal processing (PoF)
Hetal2007 ^++^ [107]	German	Age range not reported Average age: 24.6 Typical readers	Free reading RSVP	Recognition	Reading modality, Repetition	Foveal processing
KBSS2009 * [108]	German	Age range: 19–31 Average age: 23.7 Typical readers	Free reading	Reading for comprehension	Target word predictability, Semantic relatedness	Foveal processing, Parafoveal processing (PoF)
SHL2009 ^+^ [109]	Swedish	Age range not reported Average age: 27.5 Typical readers	Priming	Semantic association judgment	Parafoveal preview, Visual field of presentation	Parafoveal processing (PoF)
DSHJK2011 * [26]	German	Age range: 17–37 Average age: 23.0 Typical readers	Free reading	Reading for comprehension	Target word predictability	Foveal processing
DKS2012 ^++^ [110]	German	Age range: 19–36 Average age: 24.4 Typical readers	Boundary, Free reading	Semantic decision	Parafoveal preview, Semantic relatedness, Repetition	Foveal processing, Parafoveal processing (PoF, Preview benefit)
HLSR2013 ** [111]	English	Age not reported Typical readers	Free reading	Reading	Text type	Foveal processing
Hetal2013 ^++^ [112]	German	Age range not reported Average age: 24.0 Typical readers	Boundary, RSVP fixed-pace, RSVP self-pace	Recognition	Parafoveal preview, Reading modality, Repetition	Foveal processing, Parafoveal processing (Preview baseline)
KSS2015 * [113]	English	Age range: 18–29 Average age: 20.3 Typical readers	Free reading	Reading for comprehension	Target word frequency, Target word predictability	Foveal processing, Parafoveal processing (PoF)
MvdMVR2015 * [104]	German	Age range: 19–34 Average age: 25 Typical readers	Free reading, RSVP	Reading for comprehension	Target word predictability	Foveal processing, Parafoveal processing (PoF)
KNSD2016 ^++^ [114]	German	Age range: 18–34 Average age: 24.8 Typical readers	Boundary, RSVP-with-flankers	Semantic decision	Parafoveal preview, Reading modality, Pretarget word frequency	Foveal processing, Parafoveal processing (Preview benefit)
LPDHCB2016 ^+^ [115]	Spanish	Age range: 18–29 Average age: 20.0 Typical readers	Priming, Boundary	Semantic association judgment	Preview semantic relatedness, Target semantic relatedness	Parafoveal processing (PoF, Preview benefit)
MvdMVR2016 * [116]	German	Age range not reported Average age: 25.0 Typical readers	Free reading, RSVP	Sentence well-formedness judgment	Syntactic/semantic violations, Violation position, Reading modality	Foveal processing
ND2016 ^++^ [117]	German	Age range: 18–34 Average age: 26.1 Typical readers	Boundary, RSVP-with-flankers	Semantic decision	Parafoveal preview, Foveal load, Target word frequency	Foveal processing, Parafoveal processing (PoF, Preview benefit)
WKV2016 * [118]	Hungarian	Age range: 20–26 Average age: 22.3 Typical readers Fast and slow readers	Free reading	Reading for comprehension	Inter-letter spacing, Reading ability	Foveal processing
LHHL2018 * [119]	Finnish	Age range: 12–13.5 Average age not reported Typical readers	Free reading	Sentence plausibility judgm0nt	Semantic violations	Foveal processing, Parafoveal processing (PoF for EM data only)
DLZZDL2019 * [120]	English	Age range: 18–26 Average age: 19.3 Typical readers	Boundary	Reading for comprehension	Parafoveal preview, Target word frequency	Foveal processing, Parafoveal processing (PoF, Preview benefit)
DLZZDL2019 * [121]	English	Age range: 18–26 Average age: 19.3 Typical readers	Boundary	Reading for comprehension	Inter-word spacing, Parafoveal preview	Foveal processing, Parafoveal processing (Preview benefit)
LHHL2019 * [122]	Finnish	Age range: 12–13.5 Average age not reported Slow and typical readers	Free reading	Sentence plausibility judgment	Word length, Fixation order, Reading ability	Foveal processing

Note: The only other co-registration study to date that has used a reading task is [123]. However, the study focused on issues related to problem solving rather than aspects of linguistic processing. For this reason, this study is not discussed in the present review.

**Table 2:** Summary of the Findings Reported in Co-Registration Studies Investigating Parafoveal-on-Foveal (PoF) Effects. In these studies, the parafoveal word was manipulated, and the eye movements (EM) and fixation related-potentials (FRP) data were time-locked to the fixation onset on the pretarget word. Thus, these results are associated with effects derived from parafoveal manipulations measured during fixation on the pretarget word.

Investigated Effect	Study	FRP Data				EM Data		
		Significant	Time-Window	Electrode Sites	Direction of the Effect	Significant	EM Measure	Direction of the Effect
Word form	BM2005 [106]	yes	peak 119 ms (N1)	LO	related words > letter-string	yes	TFD	words > letter-string
			from 100 ms, peak 140 ms (P)	RC, RF	unrelated words > letter-string			
	SHL2009 [109]	yes	200–280 ms (P2)	O	RVF words > RVF letter-string	yes	FFD	RVF unrelated words > letter-string
							TRT	words > letter-string
	KNSD2016 [114]	no	200–280 ms (N1)	OT		not analysed		
	DLZZDL2019 [120]	yes	70–120 ms (P1)	RO, RP, MO, MP	X-string > identity	yes	(FFD)	X-string > identity
				RO, RP, MO, MP	X-string > letter-string		SFD	X-string > identity
				LO, LP, T, F	letter-string > X-string		GD	X-string > identity and letter-string
				C	identity > letter-string			
			120–300 ms (N1)	RO, RP, MO, MP	identity > X-string			
				RO, RP, MO, MP	letter-string > X-string			
				LO, LP, T, F	X-string > letter-string			
				C	letter-string > identity			
				F, C, T	X-string > identity			
			300–500 ms (N400)	RO, RP, MO, MP	identity > X-string			
				RO, RP, MO, MP	letter-string > X-string			
				LO, LP, T, F	X-string > letter-string			
				C	letter-string > identity			
				F, C, T	X-string > identity			
Word repetition	DKS012 [110]	no	from 0–40 ms to 560–600 ms	all	X	yes	FFD	repeated < different
							SFD	repeated < different
							GD	repeated < different
Preview frequency	KSS2015 [113]	no_†_	from 150–200 to 350–400 ms (N400)	CP, M	X	no	LFD	X
	ND2016 [117]	yes	130–140 ms (P)	RF	LF > HF	yes	GD	LF > HF
			630–640 ms (P)	LP	HF > LF			
	DLZZDL2019 [120]	no	70–120 ms (P1), 120–300 ms (N1), 300–500 ms (N400)	all	X	no	FFD, SFD, GD	X
Semantic relatedness	BM2005 [106]	yes	from 160 ms, peak 215 ms (P2)	C, F	related words > unrelated words	yes	TFD	unrelated words > related words
	SHL2009 [109]	no	90–140 ms (P1), 140–200 ms (N1), 200–280 ms (P2)	O	X	no	FFD, GD, TRT	X
			70–120 ms (N1), 140–280 ms (P2)	FP	X			
	KBS2009 [108]	yes	250–400 ms (N400)	P, C	unpredicted unrelated > predicted antonym	no	LFD	X
				P, C	unpredicted unrelated > unpredicted related			
	DKS2012 [110]	no	from 0–40 ms to 560–600 ms	all	X	no	FFD, SFD, GD	X
	LDHB2016 [115] *	yes	400–550 ms (N400)	all	unrelated > related	no	FFD, GD	X
Preview predictability	KBS2009 [108]	no	250–400 ms (N400)	P, C	X	no	LFD	X
	DSHJK2011 [26]	no	300–500 ms (N400)	X	X	not analysed	X	X
	KSS2015 [113]	_†_	from 150–200 to 350–400 ms (N400)	CP, M	X	no	LFD	X
	MvdMVR2015 [104]	+	334–826 ms, peak 608 ms (N) peak 658 ms (P)	CP F	incongruent > congruent incongruent > congruent	no	FFD, GD, RP	X

Note: * ERPs were time-locked to the onset presentation of the prime-preview pair. _†_Some short time-windows did show significant effects, but the authors disregarded those effects as meaningless. + The authors observed significant differences but pointed out that they could actually reflect an effect in response to the target word. RVF = right visual field; C = central; CP = centro-parietal; F = frontal; LO = left occipital; LP = left parietal; M = midline; MO = midline occipital; MP = midline parietal; O = occipital; P = parietal; FP = fronto-parietal; RC = right central; RF = right frontal; RO = right occipital; RP = right parietal; T = temporal; FFD = first fixation duration; GD = gaze duration; LFD = last fixation duration; RP = regression probability; SFD = single fixation duration; TFD = total fixation duration; TRT = total reading time; HF = high frequency word; LF = low frequency words. “>” = amplitudes associated with the left-hand side conditions were greater than amplitudes associated with conditions on the right-hand side of the symbol (i.e., more negative if a negative (N) component was observed in that time-window, more positive if a positive (P) component was present in that time-window). Fixation durations associated with the left-hand side conditions were longer (or there was an increased regression probability) than the fixation durations associated with conditions on the right-hand side of the symbol. “<” = amplitudes associated with the left-hand side conditions were lower than amplitudes associated with conditions on the right-hand side of the symbol (i.e., less negative if a negative (N) component was observed in that time-window, less positive if a positive (P) component was present in that time-window). Fixation durations associated with the left-hand side conditions were shorter (or there was a reduced regression probability) than the fixation durations associated with conditions on the right-hand side of the symbol.

**Table 3:** Summary of the Findings Reported in Co-Registration Studies Investigating Preview Effects. In these studies, the effects associated with parafoveal manipulation were measured when both EMs and FRPs were time-locked to the initial fixation onset on the target word, to examine the influence that the pre-processing of an upcoming word in the parafovea exerts on the processing of that word when currently fixated.

Investigated Effect	Study	FRP Data				EM Data		
		Signicant	Time-Window	Electrode Sites	Direction of the Effect	Signicant	EM Measure	Direction of the Effect
Identity parafoveal preview	DKS012 [110]	yes	200–240 ms (N1)	OT	invalid > identity	yes	FFD	invalid > valid previews
			240–280 ms (N1)	OT	invalid > identity		SFD	invalid > valid previews
			360–400 ms (N400)	CP	invalid > identity		GD	invalid > valid previews
	KNSD2016 [114]	yes	200–280 ms (N1)	OT	X-string > 1 letter	yes	FFD	X-string > 3 letters
					X-string > 2 letters			X-string > full preview
					X-string > 3 letters			
					X-string > full preview			
			400–500 ms (N400)	CP	invalid > identity			
	ND2016 [117]	yes	140–200 ms (N1)	OT	invalid > identity	yes	FFD	invalid > valid previews
			200–300 ms (N1)	OT	invalid > identity		SFD	invalid > valid previews
							GD	invalid > valid previews
	LDHB2016 [115]	yes	300–500 ms (N400)	not reported	invalid > identity	not analysed	X	X
			500–800 ms (P600)	C	invalid > identity			
	DLZZDL2019 [120]	yes	0–70 ms (N)	RO, MO, RP, MP	identity > X-string	yes	FFD	invalid > valid previews
				RO, MO, RP, MP	letter-string > X-string		SFD	invalid > valid previews
				RO, T, P	identity > letter-string		GD	invalid > valid previews
				C	letter-string > X-string			
				C	letter-string > identity			
				T, F	X-string > letter-string			
			70–120 ms (P1)	RO, RP, MO, MP	X-string > identity			
				RO, RP, MO, MP	X-string > letter-string			
				RO, T, P	letter-string > identity			
				C	X-string > letter-string			
				C	identity > letter-string			
				RT, RF	letter-string > X-string			
			120–300 ms (N1)	O, P (120–200 ms)	identity > X-string			
				O, T, P (200–300 ms)	X-string > identity			
				O, T, P	X-string > letter-string			
				O, T, P	identity > letter-string			
				T, C, F (120–200 ms)	X-string > identity			
				M, C	letter-string > X-string			
				M, C (200–300 ms)	identity > X-string			
				C	letter-string > identity			
			300–500 ms (N400)	O, T, P	X-string > identity			
				O, T, P	X-string > letter-string			
				O, P	identity > letter-string			
				M, C	letter-string > X-string			
				M, C	identity > X-string			
	DLZZDL2019 [121]	yes	0–70 ms	X		yes	FFD	invalid > valid previews
			70–120 ms (P1)	O, P	string > identity		SFD	invalid > valid previews
				C, F	identity > string			
			120–300 ms (N1)	O, RT, LT, RP, LP (120–180 ms)	identity > string		GD	invalid > valid previews
				C, F (120–180 ms)	identity < string			
				O, RT, LT, RP, LP (185–300 ms)	identity < string			
				MP, C, LF, MF (185–300 ms)	identity > string			
			300–500 ms (N400)	O, LP, LC, MC	identity > string			
				RT, RC, MC, F	identity < string			
Semantic relatedness	DKS2012 [110]	no	from 0–40 ms to 560–600 ms	all	X	no	FFD, SFD, GD	
	LDHB2016 [115]	yes	0–200 ms (N)	O, P, C	unrelated > related	no	FFD,	
			300–500 ms (N400)	all	unrelated > related		GD	
			500–750 ms (P600)	all	unrelated > related			

Note: OT = occipito-temporal; RT = right temporal. See Table 2 for a legend of the other abbreviations.

**Table 4:** Summary of the Findings Reported in Co-Registration Studies Investigating Foveal Processing from Fixation Onset on the Target Word. In these studies, both EMs and FRPs were time-locked to the initial fixation onset on the target word, to examine variables that affect processing of a word from (at least) its initial fixation onward and their time course.

Investigated Effect	Study			FRP Data			EM Data	
		Significant	Time-Window	Electrode Sites	Direction of the Effect	Significant	EM Measure	Direction of the Effect
Text type	HLSR2013 [111]	yes	75–125 ms (P1)	O, T	text > pseudotext	yes	FFD	pseudotext > text
			125–210 ms (N1)	O, T	text > pseudotext			
Inter-word spacing ^X^	DLZZDL2019 [121]	yes	0–70 ms^ (N)	LP, LO	unspaced > spaced	yes	FFD	unspaced > spaced
			70–120 ms (P1)	O, P	spaced > unspaced		SFD	unspaced > spaced
				F, LC, MC	unspaced > spaced		GD	unspaced > spaced
			120–300 ms (N1)	O, P (135215– ms)	spaced > unspaced			
				F, C, T (145205– ms)	spaced < unspaced			
				O, P (215300– ms)	spaced < unspaced			
				RC (145300– ms)	spaced < unspaced			
				F, C, MP (220300– ms)	spaced > unspaced			
			300–500 ms (N400)	X	X			
Inter-letter spacing	WKV2016 [118]	yes	120–175 ms (N)	OT, P	normal spacing >reduced and double spacing	yes	FD *	reduced > normal spacing
			155–220 ms (N)	ROT, RP	reduced > normal > double spacing			normal > double spacing
			230–265 ms (P)	ROT, P	normal spacing >reduced and double spacing		SA *	double > normal spacing
			345–380 ms (N)	LOT	normal spacing >reduced and double spacing			normal > reduced spacing
Word length	LHHL2019 [122]	yes	130–300 ms (P)	F	TP: long > short words for additional fixation	yes	FFD	long > short words
			130–300 ms (N)	O	TP: long > short words for additional fixation		GD	long > short words
			170–280 ms (N)	RO	SR: long > short words for additional fixation		REFIX	long > short words
Word frequency	KSS2015 [113]	no	from 150–200 to 650–700 ms (N400)	CP, M	X	yes	FFD	LF > HF
							GD	LF > HF
							skipping	LF < HF
	ND2016 [117]	yes	140–200 ms (N1)	OT	LF > HF	yes	FFD	LF > HF
			200–300 ms (N1)	OT	LF > HF		SFD	LF > HF
							GD	LF > HF
	DLZZDL2019 [120]	no	0–70 ms ^, 70–120 ms (P1),	all	X	yes	FFD	LF > HF
			120–300 ms (N1), 300–500 ms (N400)				SFD	LF > HF
							GD	LF > HF
Repetition (old/new)	Hetal2007 [107]	yes	250–600 ms (P)	P, C, F +	old > new	not analysed		
	DKS2012 [110]	yes	80–480 ms (N400)	CP	new > old	yes	FFD	new > old
							SFD	new > old
							GD	new > old
	Hetal2013 [112]	yes	176–390 ms (P)	P, C, F +	old > new	not analysed		
Semantic Relatedness	KBS2009 [108]	yes	450–740 ms (P600)	P	unpredicted unrelated > unpredicted related	no	FFD	X
				P, C	unpredicted unrelated > predicted antonyms			
				LC	unpredicted related > predicted antonyms			
	DKS2012 [110]	yes	160–480 ms (N400)	CP	unrelated > related	yes	FFD	unrelated > related
							SFD	unrelated > related
							GD	unrelated > related
	LDHB2016 [115]	yes	300–500 ms (N400)	all	unrelated > related	no	FFD, GD	X
			500–750 ms (P600)	all	unrelated > related			
Word predictability	KBS2009 [108]	yes	250–400 ms (N400)	RP	unpredicted related > predicted antonym	yes	FFD	unpredicted > predicted
				P	unpredicted unrelated > predicted antonym			
	DSHJK2011 [26]	yes	248–500 ms (N400)	CP	low predictable > high predictable	yes	FFD	LP > HP
							GD	LP > HP
							TSR	LP > HP
	KSS2015 [113]	yes	150–250 ms (P200)	CP, M	predictable > unpredictable	yes	FFD	LP > HP
			250–650 ms (N400)	CP, M	unpredictable > predictable		GD	LP > HP
							regressions	LP > HP
							skipping	LP < HP
	MvddMVR2015 [104]	yes	222–514 ms, peak 378 ms (N400)	ROT	incongruent > congruent	yes	FFD	incongruent > congruent
			318–626 ms, peak 476 ms (P)	F, FC	incongruent > congruent		GD	incongruent > congruent
			692–1400 ms, peak 1382 ms(P)	CP	incongruent > congruent		RP	incongruent > congruent
Syntactic & semantic violations	MvdMVR2016 [116]	yes	290–1000 ms (P600)	X	Mid-sentence syntactic violations regression trials > control	yes	FFD	violations > control
			540–1000 ms (P600)	X	Mid-sentence semantic violations regression trials > control		GD	violations > control
			24–378 ms (N400)	CP	Sentence-final syntactic violations regression trials > control		RP	violations > control
			244–1000 ms (P600)	CP	Sentence-final syntactic violations regression trials > control			
			98–392 ms (N400)	OT	Sentence-final semantic violations regression trials > control			
			412–1000 ms (P600)	CP	Sentence-final semantic violations regression trials > control			
			310–1000 ms (N)	CP	Sentence-final syntactic violations no-regression trials > control			
			336–646 ms (N)	CP	Sentence-final semantic violations no-regression trials > control			
			652–774 ms (N)	CP	Sentence-final semantic violations no-regression trials > control			
	LHHL2018 [119]	yes	167–547 ms (N)	RFEF	anomalous word neighbour > plausible	yes	FFD	anomalous word neighbour > plausible
			238–738 ms (N)	RFEF	unrelated anomalous > plausible			unrelated anomalous > plausible
			254–445 ms (N400)	LP	unrelated anomalous > plausible		GD	anomalous word neighbour > plausible
			263–447 ms (N400)	CP	unrelated anomalous > plausible			unrelated anomalous > plausible
			309–535 ms (N400)	LP	anomalous word neighbour > plausible		REFIX	anomalous word neighbour > plausible
			484–683 ms (P600)	LP	unrelated anomalous > anomalous word neighbour			unrelated anomalous > plausible
			558–899 ms (P600)	LP	unrelated anomalous > plausible			
			564–709 ms (P600)	RP	unrelated anomalous > plausible			
			648–739 ms (P600)	CP	anomalous word neighbour > plausible			
			710–899 ms (P600)	LP	anomalous word neighbour > plausible			
			739–813 ms (P600)	RP	unrelated anomalous > plausible			
			792–869 ms (P600)	CP	unrelated anomalous > plausible			
Foveal load	KNSD2016 [114]	yes	200–280 ms (N1)	OT	HF > LF	yes	FFD	LF > HF
Reading ability	LHHL2019 [122]	yes	140–250 ms (P)	C	Slow > Typical readers	yes	FFD	Slow > Typical readers
			250–300 ms (P)	O	Slow > Typical readers		GD	Slow > Typical readers
							REFIX	Slow > Typical readers

Note: * Fixation duration (FD) and saccade amplitude (SA) were calculated based on median values. + Although the effect was more pronounced on the right central and frontal scalp sites. ^ Note that effects between 0–70 m after fixation onset are, in fact, parafoveally triggered. That is, those effects are related to the processing of the stimulus that was in parafovea immediately preceding the fixation on the target word. ^X^ We have classified this effect as foveal, but strictly speaking, this manipulation involved both foveal and parafoveal change, in that the word final space was masked until the eyes moved onto the next word. FC = fronto-central; LC = left central; LOT = left occipito-temporal; OT = occipito-temporal; RFEF = right frontal eye field; ROT = right occipito-temporal; TSR = total sentence reading; REFIX = refixation probability; LP = low predictable words; HP = high predictable words. See Table 2 for a legend of the other abbreviations.
